# Is Sphingosine-1-Phosphate a Regulator of Tumor Vascular Functionality?

**DOI:** 10.3390/cancers14051302

**Published:** 2022-03-03

**Authors:** Manale Karam, Annette Ives, Christian Auclair

**Affiliations:** 1 AC Biotech, Villejuif Biopark, Cancer Campus, 1 mail du Professeur Mathé, 94800 Villejuif, France; manale.karam@ac-biotech.com; 2 AC Bioscience, Biopôle, Route de la Corniche 4, 1066 Epalinges, Switzerland; annette.ives@ac-bioscience.com; 3 Département de Biologie, École Normale Supérieure Paris-Saclay, Université Paris-Saclay, 94230 Cachan, France

**Keywords:** sphigosine-1-phosphate, pancreatic adenocarcinoma, hypoxia, patient-derived xenografts, gemcitabine, tumor growth, tumor vascular normalization

## Abstract

**Simple Summary:**

Despite substantial theoretical and experimental support for using vascular normalization as cancer therapy, effectively achieving this strategy in the clinic remains complex. In the present paper, we propose a novel potential approach for the induction of tumor vascular normalization, reduction of hypoxia, and improvement of conventional treatment in cancer patients. This approach consists of the pharmacological modulation of a patient’s plasma S1P levels which through a PDGF signaling can enhance tumor vasculature functionality and reduce hypoxia. This approach is proposed following a clinical observation in pancreatic adenocarcinoma patients and pre-clinical data in different in vivo tumor models, and is supported by a review of the literature describing the biological role of S1P in vascular functionality regulation.

**Abstract:**

Increasing evidence indicates that tumor vasculature normalization could be an appropriate strategy to increase therapies’ efficacy in solid tumors by decreasing hypoxia and improving drug delivery. We searched for a novel approach that reduces hypoxia and enhances chemotherapy efficacy in pancreatic adenocarcinoma which is characterized by disrupted blood vasculature associated with poor patient survival. Clinical significance of plasma levels of the angiogenic lipid sphingosine-1-phosphate (S1P) was assessed at baseline in 175 patients. High plasma S1P concentration was found to be a favorable prognostic/predictive marker in advanced/metastatic pancreatic adenocarcinoma patients treated by gemcitabine alone but not in patients receiving a combination gemcitabine and PDGFR-inhibitor. In pancreatic adenocarcinoma PDX models, oral administration of an S1P lyase inhibitor (LX2931) significantly increased plasma S1P levels, decreased tumor expression of the hypoxia marker (CA IX), and enhanced chemotherapy efficacy when combined with gemcitabine treatment. The direct effect of S1P on tumor oxygenation was assessed by administration of S1P onto tumor-grafted CAM model and measuring intra-tumoral pO2 using a tissue oxygen monitor. S1P increased pO2 in a tumor-CAM model. Thus, increasing plasma S1P is a promising strategy to decrease tumor hypoxia and enhance therapy efficacy in solid tumors. S1P may act as a tumor vasculature normalizer.

## 1. Introduction

It has long been known that tumor blood vessels display abnormal morphology and hence abnormal functionality. In tumors, the rapid growth of the malignant cell population is associated with overexpression of the pro-angiogenic factor VEGF leading to the development of disorganized blood vessel networks. Tumor blood vessels are heterogeneous with regard to organization, function, and structure [1].

In terms of functionality, the ability of the tumor vasculature to deliver oxygen and nutrients via blood vessels is drastically diminished. Tumor vessels are more permeable than normal vessels, they display immature traits due to poor and loose pericyte coverage, and may have a discontinuous endothelial cell lining with an abnormal basement membrane [2]. Increased vessel permeability results in aberrant osmotic forces, leading to accumulation of vascular contents and elevated interstitial fluid pressure [3]. Vascular resistance caused by irregular vessel shape and diameter leads to impaired blood flow, consequently there is often an inadequate oxygen supply to tumor cells [3,4]. The poor functionality of tumor vasculature leads to abnormal micro-environmental conditions with the occurrence of large hypoxic areas that impair therapeutic anti-cancer strategies including radiotherapy, chemotherapy [5], and immunotherapy [6]. Moreover, hypoxia favors the invasion processes and metastasis [7], and maintains a deleterious tumorigenic immune status [8]. In this context, it becomes very clear that the normalization of the tumor vasculature functionality could be the appropriate strategy to limit the deleterious effect of hypoxia and to improve chemotherapy and radiotherapy treatments.

Preclinical and initial clinical evidence reveals the potential benefits of vascular normalization as a cancer treatment strategy. Inhibiting the VEGF/VEGFR pathway has been a primary approach to tumor vessel normalization [9]. Small molecules and monoclonal antibodies targeting VEGF or VEGFR could induce transient tumor vessel normalization in preclinical models by: (1) promoting endothelial cell tightening and reducing the enlarged size and tortuosity of vessels [10,11], (2) promoting pericyte recruitment and increasing vessel maturation [3,11,12], and (3) activating metalloproteinases, remodeling the tumor matrix, and normalizing the basement membrane [3,11,12]. This would be associated with transient reduction in tumor vascular permeability [10,11,13] and interstitial fluid pressure [3,13], and increase in tumor perfusion [13], oxygenation [12], drug delivery [3,13], and radiotherapy efficacy [12]. Consistently, in clinical settings, a correlation has been observed between a “vascular normalization index” and the overall survival of glioblastoma patients [14]. Furthermore, several randomized controlled phase III trials showed that treatment with a combination of anti-VEGF monoclonal antibody with chemotherapy could significantly improve PFS compared to chemotherapy alone in metastatic breast cancer patients; however, without improving OS [15,16,17,18].

Given the transient vascular normalization induced by anti-angiogenic drugs (i.e., the vascular normalization window), a careful consideration of drug or radiotherapy scheduling is required to allow improved delivery and efficacy of chemotherapy and/or radiotherapy [13,19]. Thus, despite substantial theoretical and experimental support for using vascular normalization as cancer therapy, effectively achieving this strategy in the clinic remains complex.

In the present paper, we propose a novel potential approach for the induction of tumor vascular normalization, reduction of hypoxia, and improvement of conventional treatment in cancer patients. This approach consists of the pharmacological modulation of a patient’s plasma sphingosine-1-phosphate (S1P) levels.

We briefly describe the main arguments supporting this approach, including: (1) the literature evidencing the role of S1P in the vasculature development and function, (2) the clinical observation identifying plasma S1P concentration as a marker of favorable clinical outcome in pancreatic adenocarcinoma patients and suggesting that S1P may play a major role through a PDGF signaling in the tumor vasculature functionality, and (3) the preclinical data, in the pancreatic adenocarcinoma-patient-derived xenograft mouse model and the tumor-grafted chorioallantoic membrane (CAM) of a chicken embryo model, showing that increasing plasma S1P increases tumor oxygenation, reduces hypoxia, and enhances chemotherapy efficacy.

Increased plasma S1P could be achieved by inhibiting S1P lyase, the enzyme responsible for S1P irreversible catabolic pathway, and could lead to the development of a pharmacological, combinational, angiogenic strategy to fight hypoxia and improve cancer treatments.

## 2. Biological Properties of S1P

S1P (sphingosine-1-phosphate) is a bioactive sphingolipid metabolite that is produced by most cells, including platelets, erythrocytes, and endothelial cells [20]. Sphingosine, the precursor substrate for the synthesis of S1P, is derived by the hydrolysis of ceramide which is obtained from the sequential degradation of plasma membrane sphingolipids (glycosphingolipids and sphingomyelin). Although this occurs in various cell compartments, most sphingosine molecules are generated by degradation in liposomes. The catabolically generated sphingosine is phosphorylated by sphingosine kinases (SphK1 and SphK2) to produce S1P.

S1P levels are tightly controlled through production via SphKs, recycling back into sphingosine by sphingosine phosphatases (SGPP1 and 2) or lipid phosphate phosphatases (LPP1 and 2) and irreversible degradation through sphingosine-1-phosphate lyase (S1PL; also known as SGPL1 in mammals) into phosphoethanolamine (pEtN) and 2-hexadecanal [21].

S1P is known to play a crucial role as a signaling molecule in mammalian cells. Intracellular S1P can serve as a second messenger in signal transduction pathways which regulate cell differentiation and apoptosis [22]. S1P is also secreted and found at high concentrations in the circulation (blood and lymph). Extracellular S1P is an agonist of five different G-protein-coupled S1P receptors, designated S1P1–S1P5, which are expressed on most cells [23]. Autocrine and paracrine interactions between S1P and its receptors can modulate a wide range of physiological activities including cytoskeletal rearrangement, Ca^2+^ mobilization, cell survival, migration, immune cell trafficking, immune responses (including cytokine/chemokine production and MHC I/II presentation), phagolysosomal compartment maturation, angiogenesis, and vascular maturation [22,24,25].

## 3. S1P Signaling as Regulator of Vasculature Functionality

The development of any vascular system involves the assembly of two principal cell types—endothelial cells (ECs) and vascular smooth muscle cells/pericytes (vSMC/PC); i.e., mural cells, into different types of blood vessels [26]. All vessels begin as endothelial tubes that subsequently acquire a vSMC/PC coating. By interacting with endothelial cells, pericytes silence endothelial cell growth and provide mechanical stability to endothelial cell channels. Hence, before endothelial cells can form new branches, pericytes must first become detached. Furthermore, pericytes positioning around endothelial cell junctions form umbrella-like structures that cover gaps between endothelial cells and regulate barrier function. Owing to their contractile tonus, pericytes and vSMCs regulate blood pressure and microvascular flow and permeability [26]. In most cancers, activated pericytes have an abnormal shape, express markers of more immature, less-contractile mural cells, and are loosely associate with endothelial cells [27,28]. Pericyte abnormalities in tumor vessels contribute to vessel instability and sprouting, blood flow abnormalities, and tumor-cell breaching of the endothelial layer and dissemination.

S1P signaling, via specific S1P receptors, mainly S1PR1, and to a lesser extent S1PR2 and S1PR3, is a potent effector of vascular cell (EC, vSMC/PC) adhesion and motility that is important for blood vessel formation and maturation [29,30,31].

A key feature of S1P is its potent ability to stimulate platelet-derived growth factor-α and -β (PDGF-α/β) chain expression through activation of the small GTPase Ras and subsequent downstream extracellular signal-regulated kinases (ERK) and p38 mitogen-activated protein kinases (MAPK) [29]. PDGF-β is critically involved in the recruitment of pericytes to capillaries and in vSMC/PC cell proliferation during vascular growth. VEGF and PDGF-β play antagonist roles [32]. Endothelial cells secrete PDGF-β, that causes pericyte precursor cell proliferation and migration through activation of PDGFR-β receptor. Pericytes surround and cover early endothelial tubes. By contrast, endothelial cells in vascular sprouts release VEGF, which in turn mediates suppression of PDGFR-β signaling through the induction of VEGFR2/PDGFR-β complexes. This pathway abrogates pericyte coverage of endothelial sprouts leading to vascular instability and regression. An excess of VEGF release, as occurs in tumor microenvironments, results in vascular instability and the formation of an abnormal tumor vasculature network. In contrast, PDGF signaling improves tumor vasculature functionality through increased vessel stability [33]. Thus, by stimulating PDGF-β expression, S1P could induce pericyte recruitment to capillaries and restore pericyte coverage and stabilization of tumor vasculature.

Another mechanism of S1P in regulating angiogenesis and vascular maturation relates to its ability to promote intercellular interactions between endothelial cells and vSMCs/pericytes that are necessary for stabilization of newly formed blood vessels [30,31]. S1P signaling induces the trafficking and expression of functional N-cadherin and VE-cadherin adhesion proteins on endothelial cells, through the activation of Rac and Rho pathways, respectively [31,34]. While VE-cadherin is essential for interactions between the endothelial cells, N-cadherin is essential for cell–cell adhesion and signaling between endothelial cells and pericytes/smooth muscle cells [30,35]. Together, these cellular interactions reduce leakage and restore the stability and permeability of blood vessels.

Furthermore, S1P inhibits VEGF-induced vascular sprouting by promoting interactions between VE-cadherin and VEGFR2 that suppress VEGF signaling, restrict angiogenic sprouting, and stabilize new vascular connections [36,37].

In addition to inducing vascular maturation and stability, S1P enhances endothelial-cell survival through the activation of the ERK pathway [31].

## 4. Plasma S1P Is a Favorable Predictive and Prognostic Marker in Gemcitabine-Treated Pancreatic Adenocarcinoma Patients That Require PDGFR Activity

While in the search for new predictive/prognostic markers that may provide the basis for designing new treatment strategies to overcome resistance and improve the clinical outcome in cancer patients, we assessed the level and clinical significance of plasma S1P in advanced/metastatic pancreatic adenocarcinoma patients. Pancreatic adenocarcinoma is characterized by disrupted blood vasculature leading to hypoxia and treatment-resistance [38,39], giving pancreatic cancer the highest fatality rate of all cancers (median OS between 3 to 6 months, 5-year survival <5%) [40,41].

In this study, the biological samples (i.e., baseline plasma) came from patients enrolled in the AB07012 phase III trial—a prospective, multicenter, randomized, double-blind, placebo-controlled, two-parallel group, phase III study comparing the efficacy and safety of masitinib (a selective tyrosine-kinase inhibitor having, as main targets, PDGFR and KIT [42]), in combination with gemcitabine to placebo with gemcitabine, in patients with advanced/metastatic pancreatic cancer (IND# 68,317 EudraCT number 2008-000974-18). In this clinical trial, patients with inoperable, chemotherapy-naïve pancreatic ductal adenocarcinoma were randomized (1:1) to receive gemcitabine (1000 mg/m^2^) in combination with either masitinib (9 mg/kg/day) or a placebo. Three hundred and fifty-three patients were randomly assigned to receive either masitinib plus gemcitabine (*n* = 175) or placebo plus gemcitabine (*n* = 178). Median OS was similar between treatment-arms for the overall population, at, respectively, 7.7 and 7.1 months, with a hazard ratio (HR) of 0.89 (95% CI (0.70; 1.13)). Secondary analyses identified two subgroups having a significantly poor survival rate when receiving single-agent gemcitabine, one defined by an overexpression of acyl-CoA oxidase-1 (ACOX1) in blood, and another via a baseline pain intensity threshold (VAS > 20 mm). These subgroups represent a critical unmet medical need as evidenced from median OS of 5.5 months in patients receiving single-agent gemcitabine, and comprise an estimated 63% of patients. A significant treatment effect was observed in these subgroups for masitinib with median OS of 11.7 months in the ‘ACOX1’ subgroup (HR = 0.23 (0.10; 0.51), *p* = 0.001), and 8.0 months in the ‘pain’ subgroup (HR = 0.62 (0.43; 0.89), *p* = 0.012). Despite an increased toxicity of the combination as compared with single-agent gemcitabine, side-effects remained manageable [43].

For the purpose of the present study, samples from 175 patients (88 in the placebo + gemcitabine arm and 87 in the masitinib + gemcitabine arm) were randomly selected to assess plasma S1P levels and therefore, are representative of the advanced/metastatic pancreatic adenocarcinoma patient population. Patients characteristics, materials, and methods are described in Supplementary Materials File S1.

### 4.1. Plasma S1P Levels in Pancreatic Adenocarcinoma Patients

**Figure 1:**
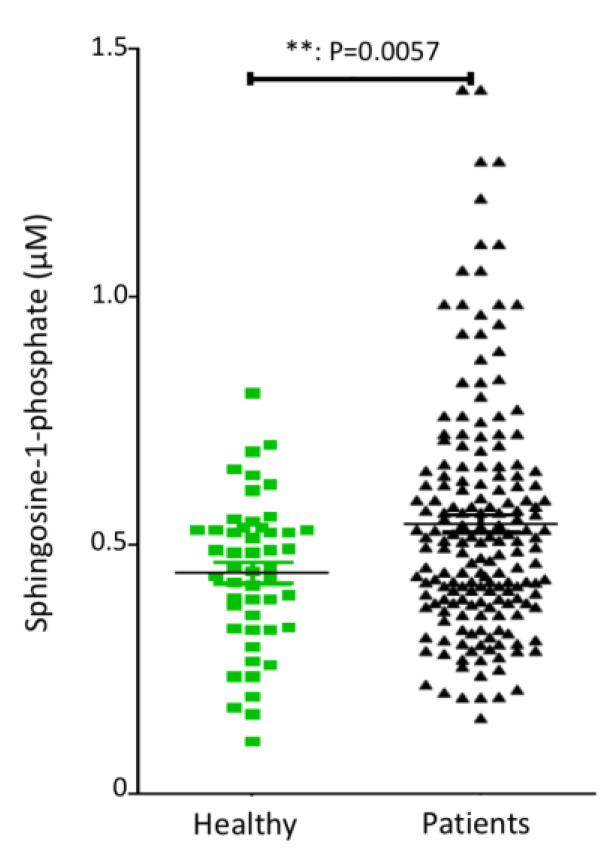
Advanced/metastatic pancreatic adenocarcinoma patients display higher baseline plasma S1P levels as compared to healthy controls. S1P concentration was determined, by ELISA, in the plasma of 50 healthy volunteers (Healthy) and 175 patients with advanced or metastatic pancreatic adenocarcinoma (Patients). The graph shows individual value plots visualizing the distribution of plasma S1P concentration in each group. The experiment was conducted, for each sample, in duplicate. The individual plots correspond to the average of the duplicate readings for each sample. The bars represent the means. Statistical significance was determined using a two-tailed, unpaired *t* test.

S1P serum levels were analyzed in 175 patients suffering from an advanced or metastatic pancreatic adenocarcinoma and 50 healthy volunteers (Figure 1). S1P serum levels ranged from 0.15 to 1.42 µM, with a median value of 0.509 µM in pancreatic adenocarcinoma patients and from 0.09 to 0.81 µM, with a median value of 0.44 µM in healthy volunteers. The difference in serum levels between patients and donors was statistically significant (*p* = 0.0057).

### 4.2. Plasma S1P Is a Favorable Predictive and Prognostic Marker in Gemcitabine-Treated Pancreatic Adenocarcinoma Patients

**Figure 2:**
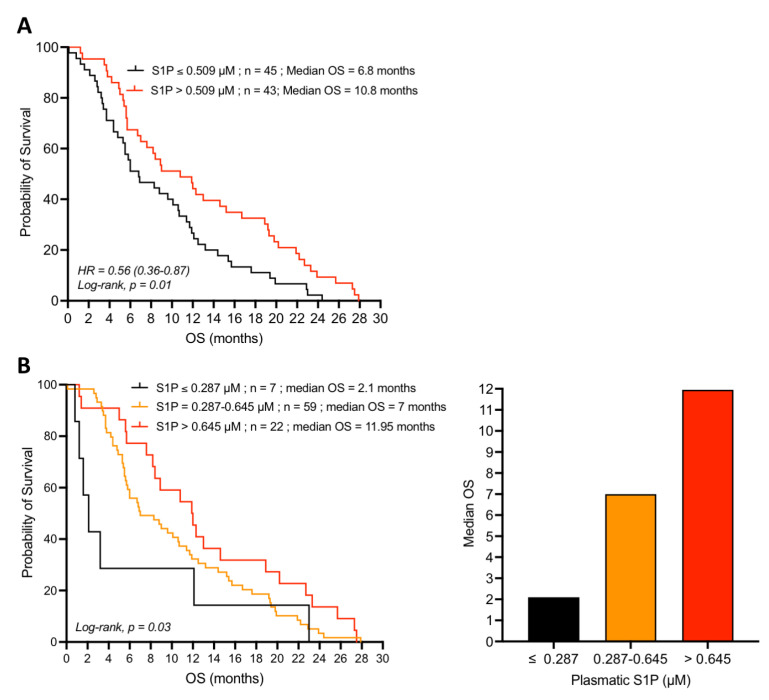
Relationship between OS and S1P plasma concentration in patients with advanced/metastatic pancreatic adenocarcinoma treated by gemcitabine. (**A**) Survival curves for patients with baseline plasma S1P levels below and above the median (0.509 µM). (**B**) Survival curves for patients with low (≤0.287 µM), intermediate (0.287–0.645 µM), and high (>0.645 µM; fourth quartile) baseline plasma S1P levels (left graph). The histogram on the right represents the median OS for each cohort. The “*n*” values represent the number of patients in each cohort.

In the group of patients receiving conventional gemcitabine treatment, those with baseline plasma S1P concentrations below the median value, i.e., 0.509 µM, displayed a poor prognosis with a median overall survival (OS) of 6.8 months. On the other hand, the subpopulation of patients with plasma S1P concentrations above 0.509 µM displayed a better prognosis with a median OS of 10.8 months (Figure 2A). Thus, patients with plasma S1P levels higher than the median (0.509 µM) had significantly longer OS than patients with plasma S1P levels below the median (HR = 0.56, 95% CI = 0.36–0.87, *p* = 0.01).

Interestingly, very low baseline plasma S1P levels (≤0.287 µM) were significantly associated with poorer prognosis and patients’ median OS following gemcitabine treatment was as low as 2.1 months (Figure 2B). In contrast, patients with intermediate (0.287–0.645 µM) and high (>0.645 µM) plasma S1P levels at baseline were more responsive to single-agent gemcitabine treatment and displayed an increased median OS, 7, and 11.95 months, respectively (Figure 2B). One-year OS was double between low (≤0.287 µM) and high (>0.645 µM) plasma S1P levels, 28% and 55%, respectively.

When using plasma S1P concentration as the continuous variable, we observed that the higher this concentration, the more responsive the patient was to gemcitabine-based chemotherapy (data not shown). High plasma S1P concentration was beneficial in terms of survival for the overall population. Thus, plasma S1P concentration appears to be a favorable prognostic marker of the clinical outcome in the patients receiving gemcitabine treatment.

### 4.3. Loss of the Prognostic Value of S1P in the Presence of the PDGFR-Inhibitor Masitinib

**Figure 3:**
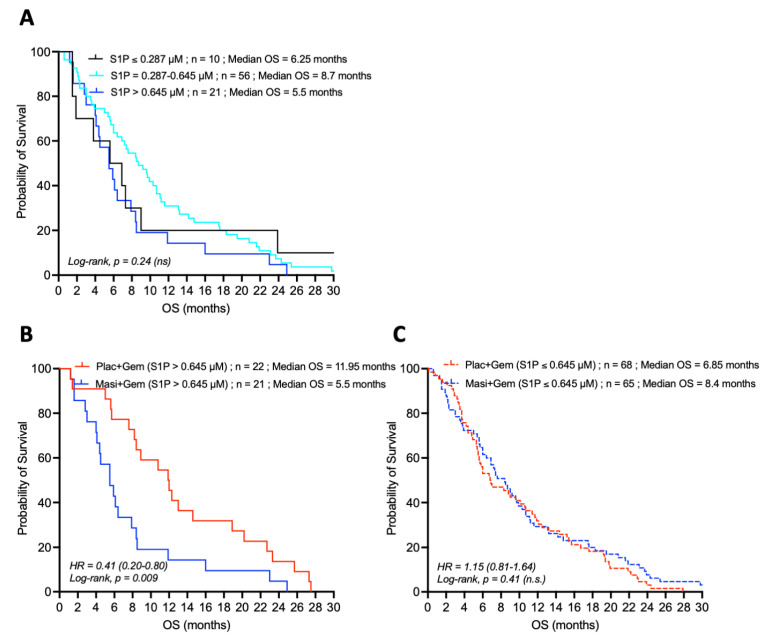
Relationship between OS and S1P plasma concentration in patients with advanced/metastatic pancreatic adenocarcinoma treated with gemcitabine and the PDGFR-inhibitor masitinib. (**A**) Survival curves for patients with low (≤0.287 µM), intermediate (0.287–0.645 µM), and high (>0.645 µM; fourth quartile) baseline plasma S1P levels and treated with the combination of gemcitabine and masitinib. (**B**,**C**) Comparative survival curves for patients with high (**B**) or low and intermediate (**C**) baseline plasma S1P concentration (above or below 0.645 µM, respectively), treated with either gemcitabine (Plac + Gem) or the combination gemcitabine and masitinib (Masi + Gem). The “*n*” values represent the number of patients in each cohort.

Similar analysis was carried out in the subpopulation of patients receiving the combination of gemcitabine plus the PDGFR-inhibitor masitinib. In contrast to the single-agent gemcitabine group (Figure 2), baseline plasma S1P levels were not significantly associated with patient OS in the masitinib and gemcitabine group (Figure 3A). Thus, plasma S1P was no longer a prognostic marker in advanced/metastatic pancreatic cancer patients treated with masitinib in addition to gemcitabine.

Comparison of the survival of patients displaying high baseline plasma S1P concentration (>0.645 µM; fourth quartile) in the two treatment groups showed that patients receiving the combination of gemcitabine and masitinib displayed a poor treatment outcome with a low median OS of 5.5 months whereas the median OS of the patients in the gemcitabine group doubled to 11.95 months (Figure 3B). On the other hand, patients with lower plasma S1P levels (≤0.645 µM) had a comparably worse prognosis whether they are treated with single-agent gemcitabine or with the combination of gemcitabine and masitinib (Figure 3C). Taken together, these data suggest that the favorable predictive value of high plasma S1P in advanced/metastatic pancreatic cancer may require the basal activity of PDGFR.

It is noteworthy that for patients with low baseline plasma S1P (≤0.287 µM) levels, those receiving the combination of gemcitabine and masitinib seemed to have an increased median OS (6.25 months) compared to those receiving gemcitabine with placebo (median OS = 2.1 months), However, this increase in median OS was not statistically significant (*p* = 0.31) (Table 1).

The identification of the favorable predictive and prognostic value of S1P, known to regulate pro-tumoral processes [22,25], in advanced and metastatic pancreatic adenocarcinoma patients treated with gemcitabine, is striking and unexpected. Regarding the S1P biological properties (described above), it can be hypothesized that S1P acts as a tumor vasculature normalizer, thus increasing drug delivery to the tumor site and hence improving the efficacy of chemotherapy. This hypothesis is supported by the results observed in the subpopulation of patients receiving the combination of gemcitabine and masitinib (Figure 3), in which plasma S1P levels were no longer associated with improved prognosis, in contrast to the subpopulation of patients receiving gemcitabine alone (Figure 2). As a potent PDGFR-α/β inhibitor, masitinib displays high anti-vascular properties and long-term use leads to a strong impairment of the tumor vascular network [33]. Thus, these effects would antagonize the action of S1P and, in turn decrease the accessibility of gemcitabine to the tumor and worsen the hypoxic status of the tumor.

## 5. Increasing Plasma S1P Levels Reduce Tumor Hypoxia and Enhance Gemcitabine Efficacy in Pancreatic Adenocarcinoma PDX-Engrafted Mice

### 5.1. Treatment with the S1P Lyase Inhibitor LX2931 Increases Plasma S1P Levels in Mice Harboring Patient-Derived Pancreatic Adenocarcinoma Xenografts

The predictive significance of plasma S1P level is of clinical interest and may open a new way for improving the treatment of pancreatic adenocarcinoma and hypoxic solid tumors. A possible strategy would be to increase the plasma S1P level prior or during chemotherapy treatment. Increase of plasma S1P level can be achieved by the inhibition of S1P lyase, an enzyme responsible for the irreversible catabolic pathway of S1P [21]. S1P-lyase inhibitors have already been described and one of them, referred as LX3305 (also known as LX2931) has been subjected to phase I and phase II clinical trials for the treatment of rheumatoid arthritis (NCT01,417,052 and NCT00,903,383).

**Figure 4:**
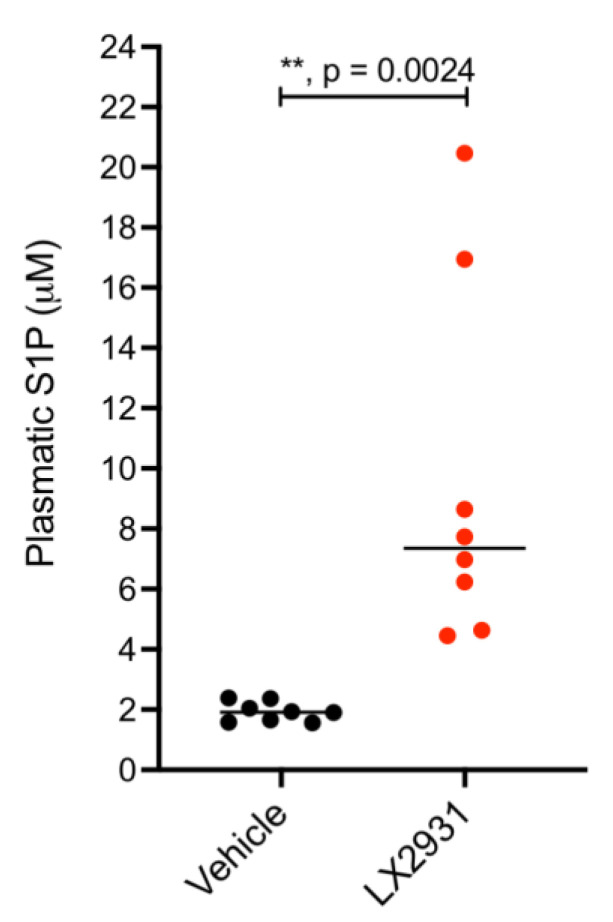
Significant increase in plasma S1P levels in mice following LX2931 treatment. Mice (Nude-Fox1nu) harboring patient-derived pancreatic cancer xenografts (PANC2-SAL) were orally treated every day for four days with 30 mg/kg LX2931 or vehicle (eight mice per group). Around 5.5 h after the last treatment, plasma was collected from retro-orbital blood and S1P plasma level was determined by ELISA assay. The graph shows individual value plots visualizing the distribution of plasma S1P concentration in each group (treated with vehicle or with LX2931). The bars represent the median. *p* value was calculated using two-tailed unpaired Student’s *t* test.

To check whether treatment with LX2931 would be a pertinent strategy to increase plasma S1P levels and subsequently assess the potential pharmacological role of S1P in improving the efficacy of gemcitabine treatment in pancreatic cancer, a patient-derived xenograft model of pancreatic adenocarcinoma was used (Supplementary Materials File S1). These mice were treated for four days with LX2931 or a vehicle control before assessment of S1P plasma levels by ELISA (Figure 4). The results show that LX2931 induced a 3.84-fold increase in median plasma S1P level.

### 5.2. Increasing Plasma S1P Levels Reduces the Expression of the Hypoxia Marker CA IX in Patient-Derived Pancreatic Tumors Implanted in Nude Mice

**Figure 5:**
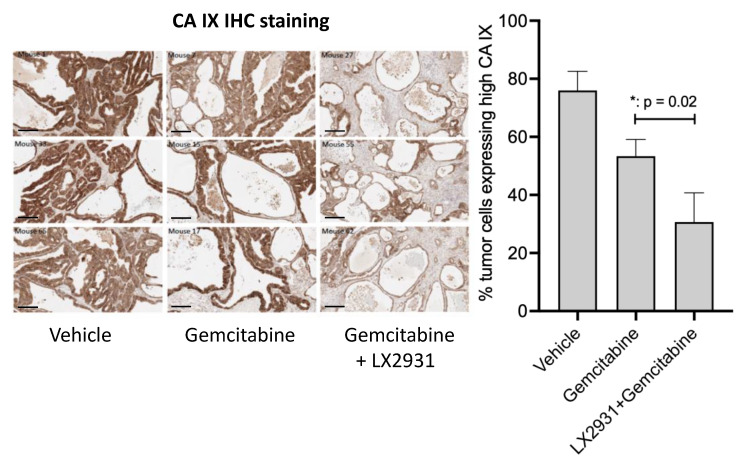
Effect of LX2931 administration on the hypoxic status of patient-derived pancreatic adenocarcinoma xenografts in nude mice. Nude mice were subcutaneously transplanted with PANC2-SAL patient-derived pancreatic cancer xenograft model. Mice bearing 62.5–220.5 mm^3^ tumors were treated with gemcitabine, the combination of LX2931 and gemcitabine or the vehicles (LX2931 and gemcitabine buffers). LX2931 was administered by p.o. route at 30 mg/kg. Gemcitabine was administered by i.p. route at 100 mg/kg. The treatment schedule was four days of LX2931 treatment, followed by one day of gemcitabine treatment and two days without treatment. This schedule was repeated for 5 weeks. At the end of study, fresh tumors from three representative mice were collected and FFPE-prepared. FFPE tumor-tissue sections were stained for the hypoxia marker CA IX by immunohistochemistry. Representative images for three tumor tissues obtained from three representative mice from each treatment group are presented. Scale bar, 200 μm. Histogram represents CA IX staining quantification ± SEM. *p* value was calculated using two-tailed unpaired Student’s *t* test.

The pharmacological relevance of the combination (LX2931 and gemcitabine) was assessed by IHC measurement of the expression of the hypoxia marker, carbonic anhydrase IX (CA IX) [44], in tumor tissues obtained from mice harboring PDXs of pancreatic cancer treated with gemcitabine or with the combination of gemcitabine and LX2931 (Figure 5). More than 75% of the pancreatic tumor cells expressed high levels of CA IX confirming the hypoxic status of this type of cancer. While gemcitabine treatment induced a 28% decrease in the number of tumor cells expressing high CA IX compared to vehicle-treated tumors, the CA IX inhibition by the combination of gemcitabine with LX2931 was much stronger (around 61%). Thus, these data suggest that the combination of LX2931 with gemcitabine reduces the hypoxic status of human pancreatic tumors in vivo.

### 5.3. Increasing Plasma S1P by LX2931 Enhances Gemcitabine Efficacy and Inhibits Patient-Derived Pancreatic Tumor Growth In Vivo

**Figure 6:**
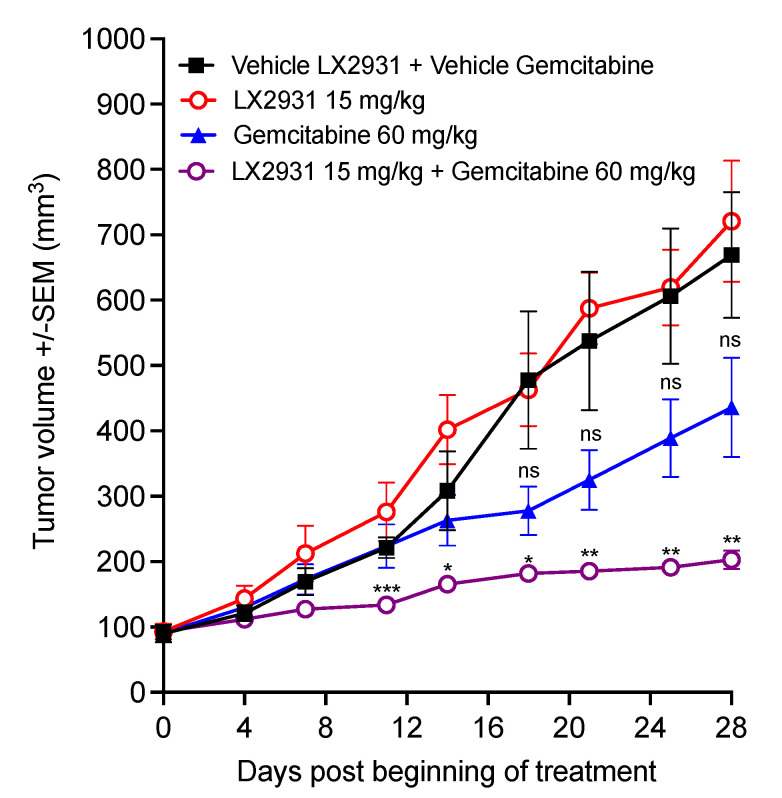
Effect of LX2931 on gemcitabine efficacy in TPAN1-IFA patient-derived pancreatic adenocarcinoma xenograft model. Nude mice were subcutaneously transplanted with TPAN1-IFA patient-derived pancreatic cancer xenograft model. Mice bearing 62.5–220.5 mm^3^ tumors were treated with gemcitabine, LX2931, the combination of LX2931 and gemcitabine, or the vehicles (LX2931 and gemcitabine buffers). LX2931 was administered by p.o. route at 15 mg/kg. Gemcitabine was administered by i.p. route at 60 mg/kg. The treatment schedule was four days of LX2931 treatment, followed by one day of gemcitabine treatment and two days without treatment. This schedule was repeated for 4 weeks. The graph shows the means of tumor volume ± SEM (*n* = 7 mice per each treatment group). *p* values were calculated using two-tailed unpaired Student’s *t* test. ns = not statistically significant, * *p* < 0.05; ** *p* < 0.01; *** *p* < 0.001 versus vehicle-treated tumors (black curve).

To evaluate whether increasing the plasma S1P level would enhance the sensitivity of pancreatic cancer tumors to gemcitabine chemotherapy, the effect of the combination of LX2931 with gemcitabine was evaluated in vivo in the TPAN1-IFA human patient-derived pancreatic adenocarcinoma xenograft model (Figure 6).

LX2931 at 15 mg/kg and gemcitabine at 60 mg/kg as monotherapy did not show statistically significant inhibition of tumor growth compared to the vehicle control (Figure 6). On the other hand, treatment with the combination of gemcitabine and LX2931 induced strong inhibition of tumor growth compared to both vehicle control and gemcitabine treatments (by 69% and 51.3% on day 24, respectively), with tumor stabilizations in all mice in combination group (Figure 6). Thus, these results show that LX2931 significantly induces human pancreatic adenocarcinoma response to gemcitabine and enhances gemcitabine efficacy in vivo.

## 6. S1P Increases Partial Pressure Oxygen (pO2) in Tumors Grafted in the Chorioallantoic Membrane (CAM) of Chicken Embryo

**Figure 7:**
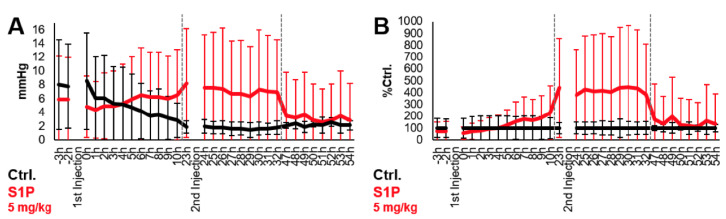
S1P increases pO2 in tumors grafted in CAM model. Human A_2780_ ovaria adenocarcinoma cells (0.25 × 106 in 33% Matrigel) were grafted onto the CAM, as previously described [45], on embryo development day (EDD) 7. Tumor-cell-engrafted CAMs were incubated at 37 °C, 65% relative humidity and atmospheric oxygen pressure 155.4 mmHg. When tumor grafts reached around 8 × 8 mm (around EDD15), 5 mg/kg S1P (*n* = 4) or vehicle control (*n* = 4) were intravenously administered twice (0 h and 14 h). Intra-tumoral pO2 was measured using Oxylite^TM^ sensors (Oxford Optronix, UK) every hour from–3 h (three hours before first S1P injection) till 54 h after first S1P injection. (**A**) Mean pO2 over time expressed in the mmHg. (**B**) Mean pO2 expressed as percentage of control (Ctrl) (i.e., CAM treated with BSA-vehicle). Gray dashed lines indicate different days of embryonal development.

The direct role of S1P in the induction of tumor vascular normalization was assessed in vivo by measuring, after direct administration of S1P molecule, the oxygenation of tumors engrafted in chick embryo chorioallantoic membrane (CAM) [45]. As shown in Figure 7, administration of 5 mg/kg S1P enhanced intratumoral oxygenation (pO2) as soon as 10 h after first S1P injection. S1P-mediated pO2 increase reached a maximum of 4-fold relative to control (tumors treated with the vehicle) at 23 h and was maintained for up to 9 h (32 h after first S1P administration). Thus, the increase in tumor oxygenation suggests that S1P enhances tumor vascular normalization.

## 7. Discussion

The benefit of antiangiogenic therapy, targeting VEGF/VEGFR, has often been observed when it is given with chemotherapy [46]. This benefit, in terms of treatment efficacy, comes from transient tumor vasculature normalization, reducing vessels leakiness and intratumoral pressure; thereby, enhancing influx of immune effector cells into tumors and improving drug delivery to tumor tissues. However, this benefit operates for only a short time, delimiting a sharp therapeutic window that is difficult to control in terms of clinical use [3,10]. Furthermore, after this therapeutic window, anti-angiogenesis therapy induces excessive pruning of vessels that has been shown to be counterproductive because it compromises the tissue delivery of drugs and oxygen, thereby increasing intratumoral hypoxia, which in turn triggers pathological angiogenesis, inflammation, radioresistance, chemoresistance, metastasis, and relapse [46,47]. Therefore, alternative approaches aimed at enhancing endothelial cell adhesion, pericyte recruitment, and interactions with endothelial cells and/or tumor matrix remodeling and basement membrane normalization without obliterating the vessels, could have a more sustained benefit.

The favorable prognostic value of S1P level in pancreatic adenocarcinoma is contradictory to the described role of S1P as a tumor promoter as revealed in the literature. In fact, S1P is known to regulate pro-tumoral signaling processes such as cell proliferation, survival, migration, inflammation, and angiogenesis [22,25] in several types of cancer including glioma and glioblastoma [48,49], ovarian cancer [50], breast cancer [51], and hepatocellular carcinoma [52]. Furthermore, previous reports assessing the prognostic significance of the expression of S1P-producing enzyme (Sphk1) or S1P receptor (S1P1) in tumor tissues revealed a significant correlation with shorter OS in NSCLC patients treated with adjuvant platinum-based chemotherapy [53], and in hepatocellular carcinoma [52] or bladder cancer patients [54], respectively. The favorable prognostic role of S1P in gemcitabine-treated pancreatic cancer, observed in the present study, may be attributed to the angiogenic, tumor vascular normalizing, and stabilizing function of S1P that could enhance tumor oxygenation and drug delivery to the tumor site, where tumor vasculature is otherwise strongly disrupted. Pancreatic adenocarcinoma is characterized by a low micro-vascular density compared to other types of cancers [38,55]. Furthermore, in contrast to other malignant tumors, the occurrence of mature tumor vasculature, as evidenced by the high expression of CD31, i.e., platelet endothelial cell adhesion molecule (PECAM-1), was identified as a favorable prognostic factor in terms of OS in patients with pancreatic adenocarcinoma [39]. Thus, discrepancies between the different cancer types could be due to a difference in the vasculature status (in relation to quantity and normality of blood vessels).

Strong evidence suggesting that the favorable prognostic value of S1P is linked to vasculature normalization is provided by the disappearance of this effect in patient receiving the combination of gemcitabine and the PDGF inhibitor masitinib. This observation agrees with the known molecular mechanism of S1P angiogenic property (as described above). Thus, by inhibiting PDGFR, masitinib would antagonize the action of S1P and, in turn decrease the accessibility of gemcitabine to the tumor. The second set of evidence comes from experimental data showing that in a pancreatic tumor PDX model and an ovarian-adenocarcinoma-tumor-engrafted CAM model, S1P decreases hypoxia marker CA IX and enhances tumor oxygenation. Importantly, increasing plasma S1P by treatment with S1P lyase inhibitor LX2931 significantly enhances gemcitabine efficacy in pancreatic adenocarcinoma PDX mice. A direct assessment of the effect of the S1P lyase inhibitor and S1P on intratumoral endothelial cell and pericyte phenotypes and interactions as well as tumor vascular perfusion would further confirm the tumor vascular normalizer property of this therapeutic approach.

Regarding the negative impact of masitinib in patients displaying high plasma S1P levels, it must be pointed out that in advanced/metastatic patients with low baseline plasma S1P (≤0.287 µM) levels, those receiving the combination of gemcitabine and masitinib seemed to have an increased median OS compared to those receiving gemcitabine with placebo. Although this would have implied that masitinib could be beneficial to patients with low S1P, this increase in median OS was not statistically significant probably due the small number of patients in each group and might require more subjects to be confirmed. Furthermore, in the AB12005 (NCT03766295) phase III trial, although masitinib in combination with gemcitabine was not overall superior to gemcitabine alone, the combination met its primary endpoint in a subpopulation of patients with a pancreatic adenocarcinoma having back pain or an overexpression of ACOX1 in blood [43]. This feature reinforces the idea that cancer treatment must, when possible, be personalized due to the heterogeneity of cancer disease leading to responder or non-responder subpopulations to the same treatment. The second consideration is that, depending on the characteristics of the cancer, combinations are not always appropriate; masitinib for instance, could be more efficient when used as single agent for the treatment of pancreatic cancer.

## 8. Conclusions

If clinically confirmed, the pharmacological modulation of S1P levels, using a S1P lyase inhibitor, may result in a more stable vasculature normalization rendering the clinical use easier and more efficient compared to VEGF signaling inhibitors. Furthermore, the feasibility and safety of this approach is supported by previous phase I and phase II clinical trials using LX2931/LX3305 for the treatment of rheumatoid arthritis (NCT01417052 and NCT00903383); thus, opening the way for the repurposing of this drug or development of other S1P modulators as tumor vascular normalizing therapies for cancer patients.

## Figures and Tables

**Table 1:** Relationship between OS and S1P plasma concentration in patients with advanced/metastatic pancreatic adenocarcinoma treated with gemcitabine alone or in combination with masitinib.

Plasma S1P Concentration	Median Overall Survival (Months)		
	Gemcitabine	Gemcitabine + Masitinib	Log-Rank *p* Value
Low (≤0.287 µM)	2.1	6.25	0.31
Intermediate (0.287–0.645 µM)	7	8.7	0.33
High (>0.645 µM)	11.95	5.5	0.009

## Data Availability

Data supporting reported results can be found in the Supplementary Materials of this article or on request from the corresponding author.

## Supplementary Materials

The following supporting information can be downloaded at: https://www.mdpi.com/article/10.3390/cancers14051302/s1, Supplementary Materials File S1. Materials and Methods.
