# Supplementary material for: Is Sphingosine-1-Phosphate a Regulator of Tumor Vascular Functionality?
Source: Cancers (Basel). 2022 Mar 3;14(5):1302. doi: 10.3390/cancers14051302 (PMC8909747; doi:10.3390/cancers14051302)
Supplement: Supplementary file 1 [file cancers-14-01302-s001.zip › cancers-1600269-supplementary.pdf]

**Supplementary Materials File S1**

## MATERIALS AND METHODS

### Patients and serum sample collection

Patients included in the trial NCT00789633 were assessed. This study consists of a multicenter, randomized, double-blind, placebo-controlled phase III trial evaluating the safety and efficacy of masitinib+gemcitabine against placebo+gemcitabine in chemotherapy-naïve advanced or metastatic pancreatic adenocarcinoma patients. A total of 353 patients from 73 active centers (located in France, the United states and the Czech Republic) were randomly assigned to receive masitinib+gemcitabine (n=173) or placebo+gemcitabine (n=176) [1]. Clinicopathologic and molecular characteristics of these patients are described in Deplanque et al 2015 [1].

Participants in the masitinib+gemcitabine arm received masitinib (9 mg/kg/day), given orally twice daily, plus gemcitabine at 1000mg/m$^2$ by intravenous (IV) infusion for 30 minutes, once every 7 days, for up to 7 weeks, followed by a week of rest. Subsequent cycles consisted of an IV infusion, once every 7 days, for 3 consecutive weeks out of every 4 weeks, until disease progression, death, limiting toxicity or patient consent withdrawal.

Participants in the placebo+gemcitabine arm received matching placebo instead of masitinib.

Patient overall survival (OS) is defined as time in months from the randomization date to the date of death due to any cause. OS was assessed up to 60 months.

Blood samples were obtained from each patient at baseline (before treatment) and centrifuged within 1 h of collection at 3600 x g for 10 min. Serums were aliquoted and stored at -80°C. For the purpose of the present study, samples from 175 patients (88 in the placebo+gemcitabine arm and 87 in the masitinib+gemcitabine arm) were randomly selected to assess plasmatic S1P levels and therefore, are representative of the advanced/metastatic pancreatic adenocarcinoma patient population.

Healthy control serum samples (n=50) were obtained from Sera Laboratories International (West Sussex, UK).

This study was approved by national health authorities and local ethics committees. Written informed consent was obtained from included patients, and patient confidentiality was protected throughout the study.

### Animals

Female athymic nude mice (*Hsd:Athymic Nude-Fox1$^{nu}$*), 6- to 9-week-old on the first day of the experimental phase, were provided by *ENVIGO* (Gannat, France).

Animals were maintained in specific pathogen-free animal housing at the Center for Exploration and Experimental Functional Research (CERFE, Evry, France) animal facility where they were delivered at least 7 days before the experiment for acclimatizing to environmental conditions.

The authorization to use animals in the CERFE facility was obtained by The Direction Départementale de la Protection des Populations, Ministère de l'Agriculture et de l'Alimentation, France *"Direction of the Veterinarian Services, Ministry of Agriculture and Food, France"* (agreement No. D-91-228-107). The animal care, housing and experiments were in accordance with French regulatory legislation concerning the protection of animals used for scientific purposes and a currently valid license for experiments on vertebrate animals, issued by the French Ministry of Higher Education, Research and Innovation, and under the supervision of authorized investigators.

### Human tumor xenograft models

Two human pancreatic adenocarcinoma PDX models (PANC2-SAL and TPAN-1-IFA) were used in this study (Xentech, Evry, France). The tumor samples were obtained from patients during surgical resection with their informed consent and established, without prior *in vitro* culture, as transplantable xenografts by subcutaneous implantation of a tumor fragment into the scapular area of immunodeficient nude mice and sequential transplantation.

**Test compound formulation and administration**

LX2931 (batch #ACB-UCB-0419) compound was manufactured at the department of chemistry at University British Columbia (Canada) and stored at 4°C, light protected. For treatment doses of 15 mg/kg and 30 mg/kg, dosing solutions were freshly prepared by solubilizing LX2931 in deionized sterile water at 1.5 mg/mL and 3 mg/mL, respectively. Preparations were stored at room temperature, in a light protected cabinet and used within 3 hours after preparation.

Gemcitabine (batch #JL5358) stock solution at 40 mg/mL was purchased from Sandoz (Levallois-Perret, France) and stored at 4°C, light protected. For treatment doses of 60 mg/kg and 100 mg/kg, gemcitabine solution was freshly diluted in NaCl 0.9% at 6 mg/mL and 10 mg/mL, respectively. Preparations were stored at room temperature, in a light protected cabinet and used within 3 hours after preparation.

Treatment consisted of oral administration of LX2931 every day for four days, followed by intraperitoneal administration of gemcitabine for one day, followed by two days without treatment. This treatment schedule was repeated for 4-5 weeks as indicated.

Vehicle controls consisted of the diluents of LX2931 and gemcitabine; i.e. deionized sterile water and NaCl 0.9%, respectively and were administered to the control mice at the same volume and schedule as their respective test compounds.

**Tumor growth assays**

For therapeutic experimental assays, mice received a subcutaneous graft of tumor fragments of around 20 $mm^3$ volume. Tumors appeared at the graft site around 4 (TPAN1-IFA PDX model) or 9 (PANC2-SAL PDX model) weeks later. Mice bearing growing tumors with a volume of 60–200 $mm^3$ were individually identified and randomly assigned to the control or treatment groups (7-8 animals/group). Treatment started at day 1. Tumor volumes were evaluated twice a week by measuring perpendicular tumor diameters with a caliper. Each tumor volume was calculated according to the following formula:

Tumor volume ($mm^3$) = [length (mm) x width $(mm)^2$]/2, where the length and the width are the longest and the shortest diameters of the tumor, respectively.

Mice were sacrificed at the end of the treatment period or when they reached any of the following ethical criteria: general alteration of behavior or clinical signs; tumor volume ≥1764 $mm^3$, body weight loss (BWL) of ≥20% or ≥15% maintained for 48 hours at any time compared to the weight on the day of enrollment (if last recorded tumor volume ≤1100 $mm^3$), BWL of ≥15% or ≥10% maintained for 48 hours at any time compared to the weight on the day of enrollment (if last recorded tumor volume ≥1100 $mm^3$). Animal diet consisted of AIN93G Nude -B6 pellets (SAFE SAS, Augy, France). Mice were offered sterilized tap water ad libitum throughout the study.

**Blood sampling**

120-130 µl of blood was collected from PDX mice by retro orbital puncture under isoflurane anaesthesia. Blood is transferred into BD Microtainer Hep-Li (200-400µl; ref: BD365966), then centrifuged at 5000 rpm at room temperature for 5 min. At least 50 µL of plasma are collected in polypropylene tubes and frozen (-20°C ± 5°C) until ELISA testing.

**Tumor sampling, fixation and paraffin-embedding**

Fresh tumors from PDX mice were fixed in 10% formalin for 24 hours and transferred in ethanol 70%, and then sent to Althisia Histology Labs (Troyes, France) for paraffin embedding. The duration of tumor storage in ethanol 70% did not exceed 2 weeks. The samples were dehydrated using ethanol and xylene and impregnated in paraffin (3 baths at 65°C in vacuum atmosphere on Peloris (Leica). After processing, the samples were embedded in paraffin blocks (Diawax paraffin).

**Immunohistochemistry Staining**

Formalin-Fixed, Paraffin-Embedded (FFPE) tumor sections (5 µm) from PANC2-SAL PDX tumors were used to determine the expression of CAIX by immunohistochemistry using CAIX polyclonal antibody (dilution 1:1000) (NB100-417SS; Novus Biologicals). The staining was performed by HistoWiz (New York, USA) and stained sections were scanned with the Leica Aperio AT2 scanner.

**Enzyme-linked immunosorbent assay (ELISA)**

S1P levels were measured in plasma samples from patients or PDX mice as indicated, using competitive ELISA (Sphingosine-1-Phosphate Assay Kit; #K-1900; from Echelon Biosciences Inc, Utah, USA), according to manufacturer's specifications. Briefly, samples were diluted 1/10 in delipidized serum. Diluted samples and S1P standards were combined with anti-S1P antibody (Clone #LT1002) and the mixtures were transferred in duplicates to the wells of blocked S1P-coated microtiter plate. After 1 hour incubation at room temperature, the mixtures were removed and the plate washed 3 times with PBS. Then, Streptavidine-HRP was added to the wells. After 1 hour incubation at room temperature, streptavidine-HRP was removed and the plate was washed 3 times with PBS. The substrate solution (TMB) was added to the wells and incubated for 30 min at room temperature in the dark before the reaction was stopped with 1 N sulfuric acid. The absorbance of the samples was measured at 450 nm on a plate reader (Varioskan LUX Reader version 1.00.38; ThermoFisher Scientific). Standard curve and sample values were calculated using GraphPad Prism 7 software (San Diego, CA, USA).

**Statistical analysis**

Overall survival curves were estimated using the Kaplan-Meier method and values between groups were compared using log-rank test. Hazard ratios (HR) and 95% confidence interval (95% CI) were calculated with the Cox proportional hazard regression model.

For non-clinical assays, statistical significance of differences between experimental groups was determined by Student's t-test using the GraphPad Prism 7 software (San Diego, CA, USA). Differences were considered significant at confidence levels greater than 95% ($P < 0.05$).

1. Deplanque, G.; Demarchi, M.; Hebbar, M.; Flynn, P.; Melichar, B.; Atkins, J.; Nowara, E.; Moye, L.; Piquemal, D.; Ritter, D.; et al. A randomized, placebo-controlled phase III trial of masitinib plus gemcitabine in the treatment of advanced pancreatic cancer. *Ann Oncol* **2015**, *26*, 1194-1200, doi:10.1093/annonc/mdv133.
